# How Do Speech-Language Pathology Social Communication Interventions Incorporate the Strengths and Perspectives of Autistic Children and Their Families: A Scoping Review

**DOI:** 10.1177/13623613261448948

**Published:** 2026-06-03

**Authors:** Maya Albin, Peter Rosenbaum, Eniola Bode-Akinboye, Yani Hamdani, Michelle Phoenix

**Affiliations:** 1 McMaster University, Hamilton, ON, Canada; 2 CanChild Centre for Childhood-Onset Disability Research, Hamilton, ON, Canada; 3 University of Toronto, ON, Canada; 4 KidsAbility Centre for Child Development, ON, Canada

**Keywords:** autism, family-centered care, neurodiversity, rehabilitation, social communication, speech-language pathology, strength-based care

## Abstract

**Purpose:**

It is important to understand how social communication interventions for autistic people align with neurodiversity-affirming approaches, including strength-based and family-centered care principles. In this scoping review, we explored how the strengths and perspectives of autistic children and their families are included in speech-language pathology social communication interventions. We searched OVID Medline, Embase, PsycINFO, and Web of Science databases, used supplementary search methods, and conducted a gray literature search. Data were extracted using the Population, Concept, and Context framework for scoping reviews.

**Major findings:**

26 articles were included in our analysis. Most studies described only deficits associated with autism. Most studies explicitly reported parents’ perspectives on intervention goals, activities, or outcome measures; children’s perspectives were rarely included. Most speech-language pathology documents from the gray literature recommended strength-based, and family-centered service delivery.

**Conclusion:**

Strength-based and family-centered values have been recommended in speech-language pathology practice for decades yet were not consistently reflected in social communication interventions for autistic children. Our discussion offers several suggestions for taking a strength-based approach to speech-language pathology practice and advancing child and family involvement toward shared decision-making. Our ideas may prompt speech-language pathology researchers and clinicians to reflect on their own approaches to autism and social communication interventions.

**Lay Abstract/Plain Language Summary:**

**Why was this study done?**

Autistic children and youth often participate in social communication interventions. These interventions can be delivered by healthcare professionals including speech-language pathologists. It is important to find out if these interventions talk about autistic people’s strengths and if they include autistic people’s and their families’ perspectives. These principles are important to make sure that interventions are neurodiversity-affirming. To answer this question, we searched for academic articles that talked about speech-language pathology social communication interventions for autistic children and youth. We used a research methodology called a scoping review. We wanted to find out whether and how these speech-language pathology interventions included the strengths and perspectives of autistic children and their families.

**What did the researchers find?**

We included 26 articles and analyzed them. We found that most studies described only the deficits associated with autism. Most studies included the perspectives of parents in their interventions, but children’s perspectives were rarely reported. We also looked at speech-language pathology documents related to autism and found that most of these documents recommended strength-based and family-centered services.

**What are important takeaways?**

Most existing social communication interventions in the field of speech-language pathology focused on autistic people’s deficits and used person-first language (e.g., person with autism) which describes autism as a diagnosis to have rather than an aspect of someone’s identity. Our discussion about our paper suggests how researchers and clinicians can incorporate autistic people’s strengths and be neurodiversity-affirming in their interventions. We also discuss ways to involve autistic children and their families in intervention decision-making, including as co-researchers. We hope that this paper will encourage speech-language pathology researchers and clinicians to think about how they view autism, and if their interventions are neurodiversity-affirming.

Autistic^1^ children often have varied social communication preferences, skills, and experiences in play, friendship development, conversation, and non-verbal communication (American Psychiatric Association [APA], 2013; Wood-Downie et al., 2021). As a result, they often face challenges navigating a social world that is primarily built upon neurotypical social communication norms and expectations (Cresswell et al., 2019; Fox et al., 2024). Social communication interventions are routinely recommended for autistic children with the aim of supporting social communication skill development (CDC, 2024; Fuller & Kaiser, 2020; Public Health Agency of Canada, 2025); these are often delivered by speech-language pathologists, among other professionals (American Speech-Language-Hearing Association [ASHA], n.d.; Public Health Agency of Canada, 2025). Historically, social communication interventions have followed a deficit-focused medical model of autism, whereby autistic children were viewed as having social communication deficits that required remediation. Such an approach aligns with current diagnostic criteria for autism that describe “persistent deficits in social communication and social interaction” (APA, 2013), and dominant theories of autism that attribute autistic traits to deficits in theory of mind, empathy, social motivation, or other social communication concepts (e.g., Baron-Cohen et al., 1985; Chevallier et al., 2012; Smith, 2009).

Deficit-focused approaches to autism have been used for decades in healthcare as part of the medical model of disability but are criticized for ignoring autistic people’s strengths, failing to consider societal and interpersonal contributions to social communication, and causing harm to autistic people’s mental health and wellbeing (Bradley et al., 2021; Cassidy et al., 2020; Kapp, 2019). In recent years, the neurodiversity movement has shifted how we talk about autism and autistic children and youth, with autism increasingly described using a strength-based lens (Cherewick et al., 2024; Kenny et al., 2016). Strength-based approaches are not unique to autism and are foundational in fields such as positive psychology (Duckworth et al., 2005). Strength-based approaches affirm autistic pride and identity, recognize that many autistic characteristics are unique strengths, and contrast with descriptions of autism that frame all autistic characteristics as deficits (Donaldson et al., 2017; Timler & McCaslin, 2025). For example, autistic adults self-report many strengths including authenticity, strong sense of justice and fairness, humor, open-mindedness, and divergent thinking (Best et al., 2015; Kirchner et al., 2016; Samson & Antonelli, 2013). Reported strengths of autistic children include caring deeply about others and kindness (Lei & Krouzkova, 2026).

Strength-based interventions improve outcomes for autistic people across disciplines, including evidence from education (Lee et al., 2020), psychology (Cherewick et al., 2024; Taylor et al., 2023), rehabilitation (Donaldson et al., 2017), and advocacy organizations such as *Autistica* (Huntley et al., 2019). For example, Taylor and colleagues (2023) found that strength-based approaches to autism were associated with improved well-being, mental health, and quality of life of ratings, and Steiner (2010) found improved outcomes for autistic children in strength-based parent-led interventions. Strength-based approaches to intervention must be delivered thoughtfully, avoiding patronizing language, stereotyping autistic strengths (e.g., concept of “savant skills”), overlooking challenges or higher support needs, and ensuring that strengths and challenges are contextualized based on societal and environmental barriers rather than framed in opposition (Urbanowicz et al., 2019). For example, Woods and Estes (2023) contrast deficit-focused diagnostic criteria for autism (e.g., focused interests, stimming, sensory differences, and social differences) with strength-based examples of these same characteristics in contexts where they are advantageous.

Family-centered care is another value in rehabilitation and pediatric healthcare service delivery that aligns with neurodiversity-affirming care principles (Braun et al., 2017; Dwyer et al., 2025; Kapp, 2020). Family-centered care includes both relational aspects (e.g., partnership between parents and providers, strength-based approaches to care) and participatory practices such as recognizing families as experts, collaborating with families in decision-making about services, and building capacity for families to implement therapeutic techniques (Bamm & Rosenbaum, 2008; Dunst & Espe-Sherwindt, 2016). Examples of family-centered service delivery include gathering information about each child and family’s values and expectations during assessment, coaching or training parents to deliver interventions, or engaging families in intervention planning (Phoenix et al., 2020; Ziegler & Hadders-Algra, 2020). However, barriers to family-centered care remain, including a lack of education for clinicians on how to partner with families (Mandak & Light, 2018), system-level challenges such as scheduling difficulties or a lack of time (Jenner & Hopf, 2023; Mandak & Light, 2018), and attitudinal barriers, whether implicit or explicit, such as the long-held perspective of clinicians as experts above family expertise (Mandak & Light, 2018; Ziegler & Hadders-Algra, 2020).

## The Changing Landscape in Speech-Language Pathology Service Delivery

Speech-language pathology (SLP) literature is also shifting toward strengths-based, family-centered, and neurodiversity-affirming approaches that incorporate the perspectives of autistic children, youth, and their families (Boron, 2025; Donaldson et al., 2017; Gaddy & Crow, 2023; Timler & McCaslin, 2025). Both family-centered care and neurodiversity-affirming care emphasize the importance of individualizing support to the unique needs of each child and family and involving families as decision-makers in intervention. Family-centered care has been recommended for decades for healthcare and rehabilitation service delivery (American Academy of Pediatrics, 2012; Bamm & Rosenbaum, 2008; Dunst & Espe-Sherwindt, 2016; Law et al., 2005) as well as specifically for autistic children and youth (Gabovitch & Curtin, 2009; Hodgetts et al., 2013; Mandak & Light, 2018; Prelock & Hutchins, 2008). Strengths-based and family-centered service delivery models were established in the field of SLP before the neurodiversity movement was applied widely, yet their principles align well.

There is also emerging research on how speech-language pathologists navigate service delivery for autistic people in the context of the neurodiversity movement (Davies, 2024; Gaddy & Crow, 2023; SAC, 2023). For example, Davies (2024) interviewed private practice speech-language pathologists, and found that many were re-evaluating their use of terminology related to autism, their conceptualization of “rehabilitation” in the context of neurodiversity-affirming approaches, and their role for social communication interventions for autistic children (Davies, 2024). Terminology related to disability and autism is constantly evolving and impacts the lenses that healthcare professionals, such as speech-language pathologists, apply to their services (Gibson, 2019; Lamoureux et al., 2024). Other changes in language and terminology include a shift toward identity-first language (i.e., autistic child) instead of person-first language (i.e., child with autism) (Bottema-Beutel et al., 2021; Kenny et al., 2016; Taboas et al., 2023) and changes to how autistic people’s communication is described (i.e., “non-speaking” instead of “non-verbal”) (Bottema-Beutel et al., 2025). These shifts in language have emerged from autistic people’s advocacy as well as broader recognition of the harms associated with ableist language, which can perpetuate stigma and negatively impact autistic people’s mental health and wellbeing (Bottema-Beutel et al., 2021; Bottini et al., 2024; Dwyer et al., 2022).

## Moving toward Neurodiversity-Affirming Social Communication Services

There is a growing body of literature examining social communication through a neurodiversity-affirming lens. In recent years, the neurodiversity paradigm and research on topics such as the Double Empathy Problem (Milton, 2012; Milton et al., 2022) have challenged deficit-focused conceptualizations of autism (Kapp, 2019; Leadbitter et al., 2021; Singer, 1998; Williams et al., 2021). From a neurodiversity-affirming perspective, autistic people’s social communication profiles are different, not deficient; (Caldwell-Harris & Schwartz, 2013). Social communication supports should be tailored to each person’s unique environment and should focus on people’s strengths rather than seeking to change autistic people (Donaldson et al., 2017; Timler & McCaslin, 2025).

Social communication interventions for autistic children and youth have also been criticized for deficit-focused approaches and a lack of input from autistic people (Bottema-Beutel et al., 2018; Carruthers et al., 2020; Monahan et al., 2023; Santhanam & Hewitt, 2020). Few social communication interventions include the firsthand perspectives of autistic people in their development or evaluation, and most rely only on proxy reports which reduce autistic people’s agency and self-advocacy opportunities (Karni-Visel et al., 2024; Tesfaye et al., 2022). There is also evidence that some social communication interventions can directly or indirectly promote camouflaging also referred to as masking, impression management, or adaptive morphing. On one hand, camouflaging can help autistic people navigate discrimination by “fitting in.” On the other hand, it can have negative mental health impacts such as increased anxiety, depression, and suicidal ideation (Bradley et al., 2021; Cage, Troxell-Whitman, 2019; Cassidy et al., 2020; Cook et al., 2021; Pearson & Rose, 2021).

There is an important need to understand how SLP social communication interventions align with strength-based and family-centered care principles. There have been many reviews on the content and outcomes of social communication interventions for young children (Carruthers et al., 2020; Gibson et al., 2021; Pacia et al., 2022), school-aged children and youth (Reichow et al., 2012; Sutton et al., 2018), and reviews specific to SLP social communication interventions for autistic preschoolers (Binns et al., 2021). No studies, to our knowledge, have explored how the SLP social communication intervention literature describes autistic children (i.e., strength-based or otherwise), or how child and family perspectives are incorporated in intervention design or delivery (i.e., family-centered care principles). There is a growing body of literature criticizing deficit-focused approaches to social communication across disciplines (Bottema-Beutel et al., 2018; Carruthers et al., 2020; Graf-Kurtulus, & Gelo, 2025), and a need to identify more strengths-based and neurodiversity-affirming approaches to guide SLP researchers and clinicians who study and/or deliver social communication interventions for autistic children and youth.

## Objective and Research Questions

The primary objective of this scoping review was to review whether and how existing SLP social communication interventions include the strengths and perspectives of autistic children and their families to provide guidance for future intervention research, development, delivery, and evaluation. We asked two research questions to achieve our objective. In published SLP social communication interventions for autistic children and youth:

**Research Question 1:** How are autistic children and youth described?
**Research Question 2:** Do the intervention goals, activities, and outcome measures explicitly incorporate child and/or parent perspectives?

We also explored SLP professional association and regulatory body documents to understand what recommendations are explicitly listed for describing autistic children, and for incorporating child and/or parent perspectives in intervention goals, activities, or outcome measurement.

## Method

A scoping review (Arksey & O’Malley, 2005; Levac et al., 2010; Peters et al., 2020; Pollock et al., 2023) was conducted to synthesize evidence, identify research gaps, and disseminate knowledge regarding SLP social communication interventions for autistic children and youth. Scoping review principles were followed to (a) identify research objectives; (b) search the literature for relevant studies; (c) select studies systematically; (d) chart the extracted data; and (e) collate, summarize, and report the results (Arksey & O’Malley, 2005; Levac et al., 2010; Peters et al., 2020). This review is reported based on the PRISMA extension for Scoping Reviews (PRISMA-ScR) (Tricco et al., 2018). We used Pollock and colleagues’ (2023) recommendations for scoping reviews to guide data extraction, synthesis, and analysis. Search strategy and inclusion criteria documents were uploaded to Figshare, an open-access repository, in October 2024 prior to full text screening and were made public in November 2024 (https://figshare.com/s/6e008854dd4277bf1267?file=49872516 & https://figshare.com/s/8e016c56bbcbe41065f7?file=49872489).

### Development of Research Questions and Objectives

A multidisciplinary team consisting of a SLP and PhD student (MA), pediatrician and scientist (PR), and an SLP and scientist (MP) developed the research questions. MA and MP consulted with MA’s dissertation committee members, SLP and scientist (LT) and Occupational Therapist (OT) and scientist (YA), who provided feedback to refine the research and data extraction questions. All team members are Canadian neurotypical healthcare providers who have clinical experiences supporting autistic people in their roles but are not autistic themselves. Our team conducted preliminary database searches of OVID MEDLINE, OVID Embase, Cochrane, and Google Scholar; we found many reviews of social communication interventions for autistic children and youth (Carruthers et al., 2020; Gibson et al., 2021; Pacia et al., 2022; Reichow et al., 2012; Sutton et al., 2018), but no reviews focused on how the interventions described autistic children or how autistic children’s and families’ perspectives were present in interventions. The search strategy for this review built upon a previous review conducted by our team (Albin et al., 2026) and used distinct data extraction and analysis (Albin et al., 2026). Gray literature (i.e., SLP professional association and regulatory body documents) was added to the current review to situate the results from peer-reviewed literature within recommendations in the field of SLP.

### Search Strategy to Identify Relevant Studies

#### Database Search

Following discussion by MA and PR, a McMaster University health sciences librarian provided guidance and feedback on the research question, databases, and search terms. Electronic searches of OVID Medline, Embase, PsycINFO, and Web of Science were conducted in October 2024. Search terms were categorized as follows: children/youth; diagnosed with autism; social communication (e.g., social skills and pragmatic language); and SLP and were adapted for each database. See Supplementary Material 1 for an example of search terms used. Covidence was used to deduplicate, collate, and review articles (Covidence, n.d.).

#### Additional Sources of Evidence for the Current Review

Supplementary search methods included (a) hand-searching the reference lists of the included articles from the database search, and (b) searching the first 50 hits on Google Scholar using the terms “autistic” + “children” OR youth” + “social communication intervention” + “speech-language pathology/ therapy.” The reference lists of all included articles were hand searched to identify any additional articles that met inclusion criteria, using the same inclusion/exclusion criteria detailed below.

The gray literature was also searched to identify relevant SLP documents that discuss service delivery for autistic children and youth. First, we inputted various permutations of terms in Google using terms such as “speech language pathology/therapy” and the names of SLP professional associations worldwide (see Supplementary Material 2) and explored each website for relevant documents on autism. We also searched “speech language pathology/therapy” AND “professional association” OR “college” AND “autism” using Google to find any documents not already identified. We used Google’s automatic translation to translate websites into English when required. Gray literature searches were completed on 23 December 2024.

### Selection of Relevant Studies

Inclusion and exclusion criteria were developed by the study team. Two reviewers, M.A. and SLP student S.K., piloted and further refined the inclusion/exclusion criteria on 15 articles and then completed minor revisions to inclusion criteria as needed before proceeding with title and abstract screening. Studies were *included* if they had participants who (a) were children and youth between the ages of 2–21 and (b) had been diagnosed with autism or other terms used in previous versions of the *Diagnostic and Statistical Manual of Mental Disorders* (5th ed; *DSM-5*); (c) social communication was listed as an aim or outcome of the intervention; and (d) there was SLP involvement in intervention creation, delivery, and/or training. Twenty-one was selected as the upper age limit as it is the age of transition out of pediatric health and education services in our own context and based on our experience with service availability. This upper limit also allowed us to focus on children and youth literature in line with our research question. Additional criteria included peer-reviewed studies with full-texts available in English and published in or after 2001 – the year that the ICF was published (WHO, 2001). The same inclusion and exclusion criteria were applied to the hand-searching of references and Google Scholar search.

### Charting and Extraction of Data

Charting and extraction of data occurred using a Microsoft Excel spreadsheet. First, M.A., M.P., and P.R. met to discuss and revise the data extraction questions, including how to define and operationalize each data extraction section. After the entire authorship team agreed with the data extraction questions, they were piloted by M.A. and E.B.A. who extracted data on the same two articles and then discussed their results. Data extraction questions were modified slightly to add additional examples in brackets and clarify wording, but no major changes were required. The data extracted included direct quotations with a corresponding page number. Data extraction was guided by the Population, Concept, Context (PCC) framework for scoping reviews, which also guided our search strategy (Peters et al., 2020; Pollock et al., 2023). See Table 1 for specific data extracted as part of the PCC framework, including population, study, and social communication intervention data, as well as data on how child and/or parent perspectives were incorporated.

**Table 1.** Population, Concept, and Context Data Extraction Questions.

Extraction component	Extraction details
Population & study data	Information on autistic children/youth:^a^ Age Number of children in the study Co-occurring diagnoses Language(s) spoken by family Socioeconomic status and/or income Race and/or ethnicity Gender and/or sex Description of autistic children/youth Autism terminology used (i.e., person- or identity-first) Descriptions of autism/autistic children as strength-based or deficit-based Descriptions of their communication and language profile Study data Year of publication Study design
Concept data	Data from SLP social communication interventions: Intervention names If interventions are manualized Professionals delivering intervention in addition to speech-language pathologists Intervention aims stated in paper Intervention outcome measures stated in paper
Context data	Incorporation of parent/guardian and/or child perspectives^b^ in: Intervention goals (i.e., what authors explicitly list as the goals created for the intervention and how the goals were determined) Intervention activities (i.e., details on specific activities or components of interventions) Intervention outcome measures (i.e., involvement in evaluation methods, sharing of parent or child perspectives on outcomes)

a We included various options for terminology to broadly capture how studies reported demographic information, not to conflate distinct terms such as race/ethnicity, or gender/sex. ^b^ Each domain was rated “yes” or “no,” with supporting data extracted from the text.

Data were extracted by MA and EBA and each acted as first coder on half of the data and as a second coder on each other’s extracted articles (Pollock et al., 2023). MA and EBA marked any additions or disagreements in a different color to track the agreement between the two reviewers and agreed on 92% of data extracted. Most disagreements could be accounted for by data that were missed by one person and added by the second to what was already extracted (e.g., adding another quotation to provide further context, missing a page number). These additions of data were then reviewed by both M.A. and E.B.A. A third reviewer (M.P.) was available to adjudicate, but this was not required. See Figshare (2024) for the extracted data (https://figshare.com/s/1058e08b3421f1db7981)

### Synthesis of Extracted Data

#### Synthesis of Academic Articles

The descriptive *population* data (e.g., number of children and demographic information) and all *concept* data (e.g., intervention names and years) were summarized in table format with additional information described in text. The remaining *population* (i.e., how autistic people and autism are described) and *context* data (i.e., how intervention goals, activities, and outcomes incorporated child and/or family perspectives) were analyzed using inductive qualitative content analysis as a descriptive approach to organize concepts into categories (Peters et al., 2020; Pollock et al., 2023). Three phases of inductive qualitative content analysis were used to (a) prepare; (b) organize; and (c) report the thematic groupings (Elo & Kyngäs, 2008; Pollock et al., 2023). To begin, MA read and re-read the extracted data. The process was iterative, beginning with open coding to create high-level categories, and later synthesizing data into thematic groupings (Elo & Kyngäs, 2008). Open coding occurred by pasting extracted data into Microsoft Word documents and using the highlight function to identify thematic groupings, as well as memo writing. All data synthesis documents, including the open coding in Microsoft Word and data extracted in Microsoft Excel, were reviewed and discussed with the authorship team who then decided how to present the results, and discussed clinical and research implications. These discussions continued until all authors agreed on how to report the data.

#### Extraction and Synthesis of Gray Literature

After identifying relevant SLP documents in the gray literature, relevant excerpts were pasted into Microsoft Word documents. Content analysis (Elo & Kyngäs, 2008), as detailed above, was used to synthesize the recommendations for talking about autism/autistic children and involving children and families in interventions in the gray literature (i.e., strength-based care, family-centered care).

## Results

### Literature Included

#### Database Search

**Figure 1.**
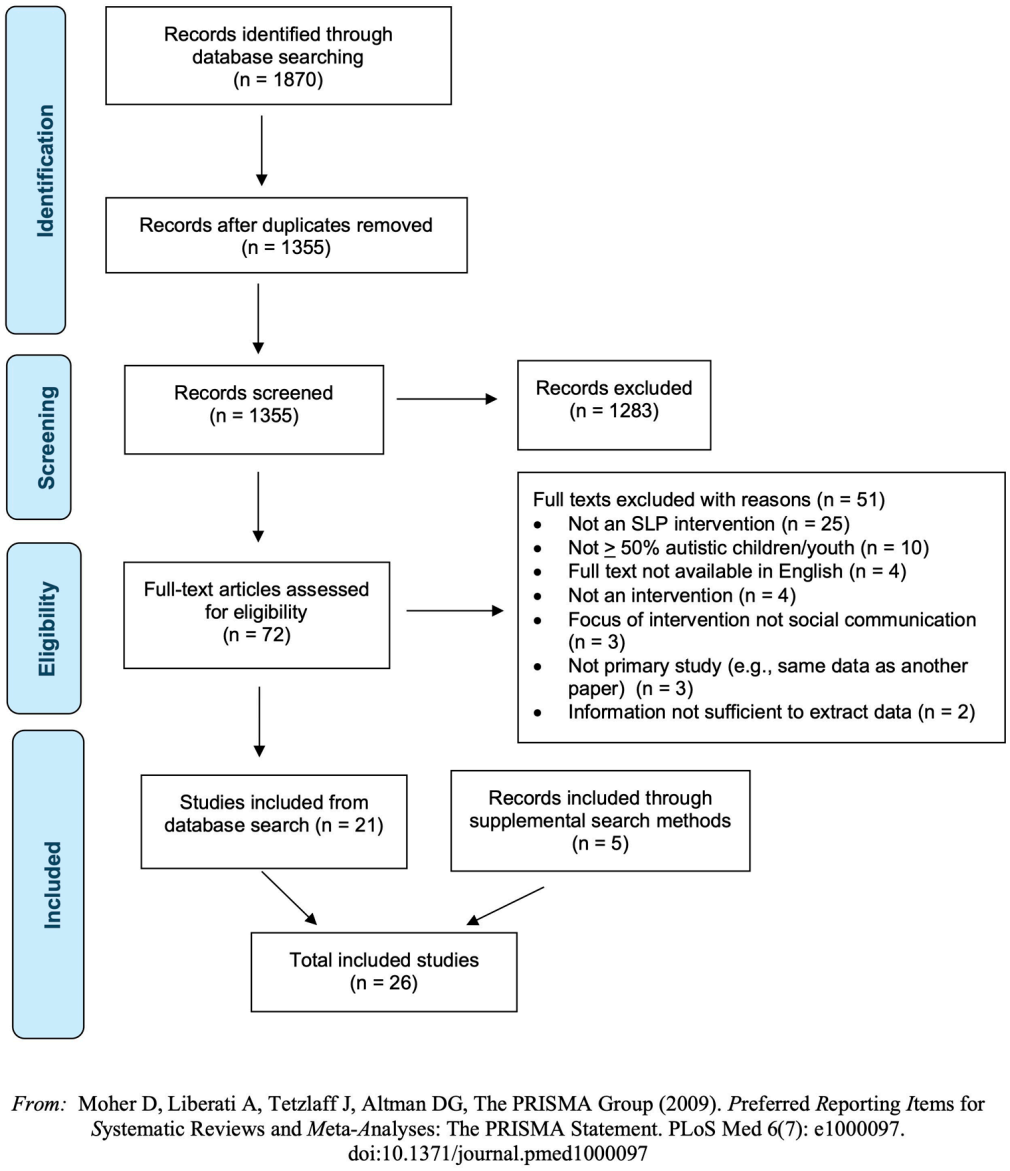
PRISMA flow diagram.

See Figure 1 for a PRISMA-ScR flow chart. The details of the database search are described in previous work by our group (Albin et al., 2026). Once duplicates were removed, 1354 titles and abstracts from the database search were screened. After screening 15 articles together to establish mutual understanding of inclusion and exclusion criteria, two reviewers (M.A. and S.K.) independently screened over 10% of all titles and abstracts to establish reliability for title and abstract screening (143 articles) and achieved high inter-rater agreement (96% agreement; Cohen’s κ = 0.65). Based on this finding, the reviewers proceeded to individually screen the remaining titles and abstracts. A third reviewer (M.P.) was available to adjudicate any disagreement throughout the screening process, which was not needed. Full texts of 72 articles were assessed for eligibility. Both reviewers screened four full texts together to ensure agreement on the inclusion and exclusion criteria, and then independently screened 10% of full texts with 100% inter-rater agreement before proceeding with individual review of the remaining full texts. Fifty-one studies were excluded with reasons listed in the PRISMA-ScR flow chart, and 21 studies were included from the database search. Supplementary search methods included (a) searching the first 50 hits on Google Scholar, which led to one article included, and (b) hand-searching the references of the 21 articles included in the database search, leading to four articles included. This, in addition to the database search, five articles were included from supplementary search methods, totaling 26 studies included.

#### Gray Literature Search

We identified five documents from SLP professional associations and regulatory bodies that discussed service provision for autistic children and youth, published between 2018-2025. The documents included: (a) Speech-Language and Audiology Canada (SAC)’s position statement on the role of speech-language pathologists supporting autistic people (SAC, 2023); (b) The American Speech-Language and Hearing Association (ASHA)’s webpage titled “Communication About Autism: Terminology Considerations” (ASHA, 2025); (c) Speech Pathology Association of Australia’s (SPA) position statement on working with autistic people (SPA, 2022); (d) The Irish Association of Speech & Language Therapists’ (IASLT) position statement on the role of speech-language therapists (SLTs) in autism (IASLT, 2018); and (e) the Royal College of Speech and Language Therapists’ (RCSLT) official guidance document on autism for speech-language therapists (RCSLT, 2023).

### Population and Study Characteristics

See Table 2 for a detailed summary of the study and population data. Studies were published between 2001 and 2024, with the largest number of studies (*N* = 7) from the United States. Seventeen studies were quantitative designs, and the remainder u either qualitative or mixed methods. 903 children participated in the interventions across studies. 25 studies had participants aged 1–12 and most included children aged 5 or younger. Only one study included participants older than age 12 (Divan et al., 2015). Seven studies reported co-occurring diagnoses in addition to autism. Twenty studies reported sex and/or gender-based information for their participants and participants were mostly male. Table 2 contains a summary of languages spoken, race and/or ethnicity, gender and/or sex, and socioeconomic status data for participants.

**Table 2.** Population & Study Data.

Citation	Child age range in years	No. of children^a^	Gender and/or sex information^b^	Languages spoken^c^	Socioeconomic status (SES) related information reported	Race or ethnicity	Country of study	Study design
Adams et al. (2020)	5–10	20	NR	NR	NR	NR	United Kingdom	Mixed
Carter et al. (2011)	1–2	62	51 boys, 11 girls	NR	Parental education levels	Breakdown of participants as White, Hispanic/Latino, Black, Alaskan Native/Alaskan Native and various combinations	United States	Quant
Casenhiser et al. (2013)	2–4	51	NR	English and “other”	Income ranges (e.g., <50k, 50–100k, 100k+)	NR	Canada	Quant
Chang et al. (2016)	3–5	38	30 males, 8 females	NR	NR	Breakdown of participants as African American, Caucasian, Hispanic, Asian, or “other”	United States	Quant
Divan et al. (2015)	5–18	17	NR	NR	NR	South Asian	India & Pakistan	Mixed
Franco et al. (2013)	5–8	6	2 males, 1 female	“English as the dominant language in the home” p. 492	NR	Breakdown of participants as Pacific Islander, Hispanic, Asian, or White	United States	Quant
Girolametto et al. (2007)	2–3	3	2 males, 1 female	NR	NR	NR	Canada	Quant
Godoy et al. (2024)	2–10	15	10 males, 5 females	Brazilian Portuguese	Monthly household income levels with Brazilian wealth index classification	Breakdown of Latino, Black & White	Brazil	Qual
Green et al. (2010)	2–11	146	132 boys, 14 girls	“Spoke English with their child” p. 2153	SES “dichotomised as at least one parent in professional or administrative occupation versus all others” p. 2154	Breakdown of parents “ethnic origin” as white, mixed, or non-white	United Kingdom	Quant
Hutchins and Prelock (2006)	6–12	2	All male	NR	NR	NR	United States	Quant
Hutchins & Prelock (2013)	4–12	20	18 males, 2 females	NR	NR	NR	United States	Quant
Katz & Girolametto (2013)	4–5	3	2 males, 1 female	NR	NR	NR	Canada	Quant
Lerna et al. (2012)	1–5	18	17 males, 1 female	NR	NR	NR	Italy	Quant
Lerna et al. (2014)	1–6	14	NR	NR	NR	NR	Italy	Quant
Lim et al. (2007)	5–7	10	All male	NR	NR	Chinese	Singapore	Mixed
Macevilly et al. (2024)	8–12	51	30 males, 21 females	English, “bilingual” p. 60	“Participants were from a range of socioeconomic classes” p. 60	Caucasian	Ireland	Quant
Miletic et al. (2024)	3–8	31	23 males, 8 females	NR	NR	NR	Canada	Mixed
Mohammadzaheri et al. (2022)	6–12	20	All male	NR	Family income rated “low” or “medium”	Iranian	Iran	Quant
Muller et al. (2016)	7–10	4	All male	NR	NR	NR	United States	Mixed
Parsons et al. (2018)	6–12	10	9 males, 1 female	Reported 8/10 English as first language	Parental education levels	NR	Australia	Mixed
Pereira et al. (2022)	3–6	20	9 males, 9 females	European Portuguese	“Same demographical region and shared similar socioeconomic backgrounds” p. 5	NR	Portugal	Quant
Salt et al. (2001)	NR	87	NR	“English-speaking households” p. 5	NR	NR	Scotland	Quant
Shire et al. (2020)	2-3	113	89 males, 24 females	NR	Program took place in “low resource neighborhoods” p. 2144	Breakdown of participants as African American, Caucasian, Hispanic, and mixed race	United States	Quant
Sun et al. (2017)	5–12	20	NR	NR	NR	NR	Brazil	Quant
Van Der Meer et al. (2014)	10	1	Male	NR	NR	NR	New Zealand	Mixed
Williams et al. (2024)	3	19	15 males, 4 females	NR	NR	NR	United Kingdom	Quant

*Note.* NR: not reported; Qual: qualitative study; Quant: quantitative study; Mixed: mixed methods study.

a We included participants that continued throughout the entire study and excluded participants who dropped out. To be included, studies needed to have at least 50% of their sample be autistic. Sample size listed includes all children that received the intervention including both autistic and non-autistic participants. For example, if a study had 9 autistic participants and 1 non-autistic participant in the intervention, the total was *N* = 10. If a study included 10 autistic participants and 10 typically developing peers as peer models (not participants in the intervention), only the 10 autistic participants were included in the total. ^b^ Language of males/females, or boys/girls was quoted from study authors and reported as it appeared in the paper. ^c^ NR indicates that languages spoken by children or families were not explicitly reported by authors. We did not make any assumptions about languages spoken, and only reported what authors explicitly stated. For example, if a study reported all participants were from Italy, but did not report specific languages spoken, we counted that as “NR” and did not infer the languages spoken.

### Social Communication Intervention Data

See Supplementary Material 3 for a summary of the social communication intervention data. Some studies reported specific intervention names, while others had general descriptions of their intervention purpose. There were 19 unique interventions across the studies; 14 studies reported manualized interventions. Most interventions were mentioned in one study, but some interventions were the focus of two or three studies, including Social Stories, Picture Exchange Communication System (PECS), Joint Attention Symbolic Play Engagement and Regulation (JASPER), Preschool Autism Communication Therapy (PACT), and More Than Words. Interventions were delivered solely by speech-language pathologists in ten studies, and the rest were delivered by speech-language pathologists and other professionals. A wide variety of social communication aims, and outcome measures, were used within and between studies, which can be found in Supplementary Material 3. Overall, most studies aimed to change perceived deficits or challenges in the child’s social communication, and some interventions focused only on parental behavior change (e.g., increased responsiveness to the child).

### What terminology and language are used to describe autistic children and their communication skills?

#### Diagnostic Descriptions of Autistic Children

See Table 3 for a summary of the terminology used to describe autistic children, and whether descriptions of autistic children were strength-based, deficit-based, or both. There was variability in the terminology used to describe autistic children across studies, reflecting both person-first and identity-first language, as well as diverse diagnostic labels and descriptors. Twenty-one studies referred to participants using only person-first language, with terms such as children “with ASD,” “with autism,” “with a diagnosis of autism,” and “on the autism spectrum.” Identity-first language (i.e., “autistic children”) was used by three studies, all published in 2024, and two studies used both person-first and identity-first language. Three studies used the term “high functioning” (Adams et al., 2020; Lim et al., 2007; Müller et al., 2016), though none explicitly defined the criteria for this label.

**Table 3.** Terminology Used to Describe Autistic Children.

Citation	Person-first (PF), identity-first (IF), or both?	Examples to support label of PF, IF, or both^a^	Strength-based (SB), deficit-based (DB), or both?	Examples to support label of SB, DB, or both^b^
Adams et al. (2020)	PF	“Children with autism spectrum disorder”	DB	“Children with high-functioning autism spectrum disorder (HFASD) have heterogeneous pragmatic deficits and long-term language processing difficulties. A related group of children, termed Social (Pragmatic) Communication Disorder (SPCD), have similar pragmatic and language impairments but may lie just below the threshold for ASD diagnosis” p. 2
Carter et al. (2011)	PF	“Children with ASD”	DB	“Communicative and social deficits are not only core to ASD, but are among the first symptoms observed” p.741
Casenhiser et al. (2013)	PF	“Children with autism,” “ diagnosed with ASDs”	DB	“Among the core symptoms of autism spectrum disorder (ASD) are deficits in abilities related to social interaction and communication . . . children with autism, for example, have been shown to display little enjoyment in interactions with others.” p. 221
Chang et al. (2016)	PF	“Children with autism spectrum disorder (ASD)”	DB	Discussing social communication interventions: “curricula lack a specific focus on the core social communication challenges experienced by children with ASD . . . challenges include joint engagement with adults and peers, joint attention gestures and language, and play skills.” p.2212
Divan et al. (2015)	PF	“Child with ASD”	DB	“The intervention focuses on addressing social, pre-linguistic, pragmatic, and linguistic impairments which are present in ASD.” p.2
Franco et al. (2013)	PF	“Children with autism”	DB	“A defining characteristic of children with autism is impairment in social interaction” p. 489. “All of the participants were classified as having moderate-to-severe levels of autism” p. 492 “Children with autism display marked deficits in initiating and maintaining social interaction . . . [and] demonstrate limited social reciprocity and decreased use of nonverbal behaviors such as eye gaze, facial expression, body postures, and gestures to communicate and regulate social interaction.” p. 489
Girolametto et al. (2007)	Both	“Children with ASD,” “diagnosed as autistic”	DB	“Autism is a developmental disorder characterized by pervasive deficits in social communication behaviour, including language, joint attention, and pragmatics” p. 471
Godoy et al. (2024)	IF	“Autistic children”	N/A	N/A – did not describe autism or autistic children in detail to extract data.
Green et al. (2010)	PF	“Children with ASD,” “with autism”	DB	“Autism is a severe, highly heritable neurodevelopmental disability . . . impairments in social reciprocity, communication, and behaviour have a profound effect on children’s social development into adulthood.” p. 2152
Hutchins and Prelock (2006)	PF	“Children with ASD” or other *DSM-5* terms	Both	Discussed a goal of “remediating the social, behavioral, and communicative impairments characteristic of autism spectrum disorders” p. 47; theory of mind as “root cause of the core deficits characteristic of ASD” Intervention supporting “strengths of persons with ASD, which typically include precocious literacy skills and a predilection to use visualization in the development of understanding” p. 48
Hutchins & Prelock (2013)	PF	“Children with ASD”	DB	Discussing interventions: “remediation of specific communicative impairments in ASD including reducing echolalia . . . interruptions . . . and loud talking”
Katz & Girolametto (2013)	PF	“Children with ASD”	DB	“Impairment in reciprocal social interaction is a core deficit in children with ASD . . . children with ASD spend less time interacting with peers than typically developing children, have lower quality interactions when they play with peers, spend more time in purposeless play or inactivity, and maintain a greater physical distance from peers . . . when these children are placed in play groups with typical peers, few or no peer interactions occur prior to intervention . . . children with ASD have significantly fewer reciprocal relationships” p. 133
Lerna et al. (2012)	PF	“Children with ASD”	DB	“Deficits of social and communication skills are core and prognostic indicators of autism spectrum disorders . . . Social – communicative impairments distinguishing ASD from typical development and developmental delay include . . . .without effective interventions, instead, these deficits tend to persist and may also lead to poor long-term outcomes and/or to . . . problematic behaviours” p. 479
Lerna et al. (2014)	PF	“Children with ASD or language delay”	DB	“Communication and social impairments appearing early in life and persisting into adulthood are core features of autism spectrum disorders” p. 610
Lim et al. (2007)	PF	“Children with ASD or language delay”	DB	“There is a general consensus that children with ASD have difficulties in social integration with their peers. There is an inability or a lack of desire to interact with their peers … Other characteristics of poor social skills include failure adequately to use eye gaze, facial expressions, body posture and gesture to regulate social interaction” p. 35
Macevilly et al. (2024)	PF	“Autistic children”	DB	Referring to autism and other diagnoses: “Children with these diagnoses may experience problems with social communication …. Social communication difficulties can exacerbate mental health difficulties, as positive social relationships generally act as a buffer against mental health disorders … social communication impairments can result in children being rejected or victimised by their peers” p. 57
Miletic et al. (2024)	IF	“Autistic children”	Both	“social communication development is a primary area of concern for caregivers of young autistic children” p. 1127, Discussing intervention: “sharing of knowledge fostered a better understanding of the autistic child’s strengths and how the family can support their development.”
Mohammadzaheri et al. (2022)	PF	“Children with ASD”	DB	“Children diagnosed with ASD exhibit challenges in receptive and expressive language, as well as in early precursors to the onset of spoken communication . . . deficits have been observed in joint attention and shared referencing . . . differences in prosody (flat intonation, poor volume control), and difficulty in verbal imitation in the early years” p. 2598 “Reciprocal social communication is one of the defining diagnostic characteristics of children with ASD . . . deficit or complete absence of social initiations . . . among children with ASD who develop verbal spoken communication, many do not initiate interactions or ask questions. A general lack social initiation toward others can interfere with the ability to establish interpersonal relationships, share mutual interests with others, and engage in reciprocal conversation” p. 2599
Muller et al. (2016)	PF	Children “on the autism spectrum,” children with “high functioning ASD”	DB	“For many children with autism spectrum disorders (ASDs), however—including those with high-functioning ASDs (HF-ASDs)—learning how to engage in spontaneous, unscripted conversation often remains maddeningly out of reach . . . These children require explicit instruction in conversation, and even then, few studies demonstrate meaningful, long-term gains” p. 191 “Due to neurological atypicalities, individuals on the autism spectrum experience significant social cognitive challenges . . . individuals with ASD lack a native ability to identify and focus on salient social stimuli, which makes it very difficult to respond quickly, flexibly, and appropriately to the ever-changing flow of social information produced during naturalistic interactions—in other words, precisely the skills required for successful conversation.” p.192
Parsons et al. (2018)	PF	“Children with ASD”	DB	“Pragmatic language and cooperative play skills are impaired in children with autism spectrum disorder (ASD) with concomitant difficulties in social interaction . . . deficits in pragmatic language are considered to be present in the language profile of all children with ASD” p.412 “Children with ASD show delays and differences in social play development . . . A common finding in research is that children with ASD have fewer friendships than their typically developing peers despite having a desire to engage in social relationships with peers” p.413 “Individuals with ASD have difficulties in the use and perception of prosody” p.416
Pereira et al. (2022)	PF	“Children with ASD”	DB	“Autism Spectrum Disorder (ASD) is a highly heterogeneous neurodevelopmental disorder . . . Pragmatic language deficits are a core feature of ASD regardless of language level or age.” p. 2
Salt et al. (2001)	Both	“Child with autism,” “autistic child”	DB	“Autism remains one of the most severe childhood psychological disorders” p. 362, “The treatment programme does not claim to be a cure for autism but prompts the early social and communication skills necessary for the child to make use of future social and educational opportunities.” p. 372
Shire et al. (2020)	PF	“Children with ASD”	DB	“Those with autism spectrum disorder (ASD) who experience core challenges in social communication and play skills that may create barriers to successful peer interactions,” “For children with ASD, engaging in reciprocal play may prove challenging both socially and cognitively due to a multitude of demands”“ p. 2143
Sun et al. (2017)	PF	“Children with ASD”	DB	“Throughout the years, despite variations in diagnostic criteria and classification, descriptions of autism spectrum disorder (ASD) include impairments in language and communication” p. 79
Van Der Meer et al. (2014)	PF	“Diagnosed by his pediatrician with ASD”	DB	“Communication impairment in ASD is characterized by the heterogeneity of expressive communication skills; with one end of the spectrum presenting as “long-winded” and talkative and the other end mute . . . although they do not have any spoken language, these individuals might be able to learn to communicate using augmentative and alternative communication.” p. 283 “ASD is characterized by persistent deficits in social interaction and social communication, including impairments in the use of multiple nonverbal behaviors such as eye-to-eye gaze, facial expression, and gestures to regulate social interaction as well as marked impairment in the ability to initiate or sustain a conversation with others” p. 2 83
Williams et al. (2024)	IF	“Autistic children”	Both	“Autism spectrum disorder (autism hereafter) is characterised by the early onset of differences in social communication and social interaction, and restricted, repetitive patterns of behaviour . . . Limited language skills are also associated with behaviour that challenges . . . poorer quality of life outcomes” “Autism has been associated with enhanced musical processing . . . including pitch discrimination and memory” p. 2516

a Examples extracted include common language used within that paper. Terms extracted capture the variation within and between papers (e.g., autistic child vs child with autism), but the examples are not exhaustive of every instance of the term in the paper. See the data extraction excel sheet on Figshare for further details. ^b^ Table includes several examples from each paper but is not an exhaustive list of every description of autism/autistic children in each paper. Authors extracted key quotes, and these were shortened further for the purpose of this table. See the data extraction excel sheet on Figshare for further details.

#### Are Strengths-Based or Deficit-Based Descriptions of Autism Reported?

Overall, descriptions of autism focused on deficits. Three studies explicitly described strengths associated with autism in addition to deficits, including strong visual learning skills, preferences for structured tasks, detailed memory, strong literacy skills, and sustained interests as skills supporting participation and learning. In 22 studies, autism was framed solely through deficits using various terms within and across studies. Many studies described children’s failure to use “expected” non-verbal communication skills in social interactions, including joint engagement and attention (Chang et al., 2016), eye gaze, facial expressions, gestures, and body posture (Lim et al., 2007; Van Der Meer et al., 2014), “nonverbal behaviors” (Franco et al., 2013, p. 489), and difficulty identifying and using social cues (Müller et al., 2016; Parsons et al., 2018). Some studies also described negative aspects of spoken communication, such as interrupting, speaking loudly, in a ‘long-winded’ way, or echolalia (Hutchins & Prelock, 2013; Van Der Meer et al. 2014; William et al., 2024; Müller et al., 2016). Some studies that described only deficits associated with autism did mention strengths related to specific participants, such as being a visual learner, a love of reading, but these descriptions were not explicitly tied to autism in the way that deficits were described as related to autism (e.g., Hutchins & Prelock, 2013).

#### Descriptions of Autistic Children’s Language Profiles

All studies included descriptions of the participants’ language skills and abilities. Descriptions were provided to contextualize a study sample, describe language gains from interventions, or as part of inclusion or exclusion criteria (e.g., participants were non-speaking, participants could not be non-speaking). Descriptions of communication primarily focused on spoken language, with three studies reporting on AAC or non-spoken communication methods: (Lerna et al., 2014; Lerna et al., 2012; Van Der Meer et al., 2014). Twelve studies discussed language delays and/or reported expressive and receptive language test scores (Adams et al., 2020; Franco et al., 2013; Girolametto et al., 2007; Green et al., 2010; Hutchins & Prelock, 2006, 2013; Katz & Girolametto, 2013; Lerna et al., 2012; Lim et al., 2007; Macevilly et al., 2024; Mohammadzaheri et al., 2022; Shire et al., 2020). Eight studies used the label of “non-verbal” or “verbal” (Adams et al., 2020; Franco et al., 2013; Green et al., 2010; Hutchins & Prelock, 2013; Lerna et al., 2014; Mohammadzaheri et al., 2022; Pereira et al., 2022; Sun et al., 2017). Four studies discussed participants lacking “functional” language or communication (Franco et al., 2013; Lerna et al., 2014; Lerna et al., 2012; Williams et al., 2024). Echolalia was described as present or absent (Williams et al., 2024), and as solitary rather than peer-directed (Müller et al., 2016).

#### What Recommendations Are Listed for Describing Autism/Autistic Children in SLP Association or Regulatory Body Documents?

SPA (2022), SAC (2023), and RCSLT (2023) used identity-first language and explained why they made this choice but did not recommend specific language to be used by speech-language pathologists. ASHA (2025) described both identity-first and person-first language as “valid and important” and encouraged clinicians to consult individual families for decisions making. IASLT (2018) used person-first language and did not discuss their reasoning. Several documents outlined how speech-language pathologists should not unnecessarily medicalize autism, and that clinicians should not aim to cure or treat autism (RCSLT, 2023; SAC, 2023; SPA, 2022). ASHA (2025) outlined how some people may view autism as a disorder to treat, while others view autism as a fundamental part of a person’s identity. RCSLT (2023) and ASHA (2025) both acknowledged how a medical model approach can be needed to facilitate access to services.

### To what extent are child and/or parent perspectives explicitly incorporated in intervention goals, activities, and outcome measures in the SLP literature?

We examined how child and/or family-centered care were taken up in intervention goals, activities and outcome measures.

#### Goals

We did not identify any studies that explicitly included children’s perspectives on intervention goals, regardless of the child’s age or communication abilities. Seventeen studies included parent perspectives on intervention goals (Adams et al., 2020; Casenhiser et al., 2013; Divan et al., 2015; Franco et al., 2013; Girolametto et al., 2007; Godoy et al., 2024; Hutchins & Prelock, 2006, 2013; Katz & Girolametto, 2013; Lim et al., 2007; Macevilly et al., 2024; Miletic et al., 2024; Parsons et al., 2018; Pereira et al., 2022; Salt et al., 2001; Van Der Meer et al., 2014; Williams et al., 2024). In most studies, parents were asked for information on their child’s strengths, challenges, and areas for growth at the beginning of the study to inform goalsetting. Examples varied widely and included speaking with parents to capture the child’s main difficulties (Godoy et al., 2024) or asking parents to complete a form that identifies three “realistic goal outcomes” (Lim et al., 2007, p. 36). Some studies had parents and therapists create mutually agreed upon goals. Examples included using the Goal Attainment Scaling (GAS) framework (Adams et al., 2020; Pereira et al., 2022), conducting a pre-study focus group with parents to determine their views (Williams et al., 2024), or discussions between parents and therapists about the child’s social communication abilities and agreeing upon goals (Divan et al., 2015; Miletic et al., 2024),

#### Activities

Two studies included children’s perspectives on activities by having children select a peer partner (Parsons et al., 2018) and select conversation topics based on their interests (Müller et al., 2016). Twenty studies mentioned parents’ input on intervention activities. Parent input was sometimes used to generalize intervention activities to “natural environments” (Divan et al., 2015; Franco et al., 2013; Lim et al., 2007; Pereira et al., 2022), such as tailoring activities to each family’s structure (Divan et al., 2015), considering the toys children had at home to apply intervention concepts (Franco et al., 2013) or ensuring that physical intervention materials were appropriate for each child (Hutchins & Prelock, 2006; Lerna et al., 2014). Parents provided input to tailor activities to the child’s developmental level, including the child’s sensory and/or physical preferences (Chang et al., 2016; Franco et al., 2013; Hutchins & Prelock, 2013; Lerna et al., 2014; Lim et al., 2007; Mohammadzaheri et al., 2022; Shire et al., 2020; Van Der Meer et al., 2014). Examples included discussion of a child’s developmental level for toy selection (Chang et al., 2016; Shire et al., 2020), tailoring activities to the child’s personal interests (Lerna et al., 2014; Van Der Meer et al., 2014) or choosing a conversation topic (Parsons et al., 2018). Activities for caregiver-led interventions were often individualized based on caregivers’ needs and preferences, such as adding activities to support caregiver mental health (Salt et al., 2001), answering parents’ questions about intervention activities and making adaptations (Casenhiser et al., 2013; Divan et al., 2015; Franco et al., 2013; Parsons et al., 2018), and analyzing videos to reflect on parents’ strategy use (Carter et al., 2011; Miletic et al., 2024; Williams et al., 2024).

#### Outcome Measures

We did not identify any studies that explicitly included children’s perspectives on intervention outcome measures regardless of the child’s age or communication abilities. Eighteen studies included parents’ perspectives on the social communication-focused outcome measures and asked parents about changes they noticed for their child during the intervention (Adams et al., 2020; Carter et al., 2011; Girolametto et al., 2007; Godoy et al., 2024; Green et al., 2010; Hutchins & Prelock, 2006, 2013; Lerna et al., 2014; Lerna et al., 2012; Lim et al., 2007; Macevilly et al., 2024; Miletic et al., 2024; Mohammadzaheri et al., 2022; Parsons et al., 2018; Pereira et al., 2022; Salt et al., 2001; Van Der Meer et al., 2014; Williams et al., 2024). For example, parents were asked to report “social skills that they found their child to have shown improvements in” (Lim et al., 2007, p. 37), impressions of change or lack thereof using a Likert-type-scaled tool (Hutchins & Prelock, 2013), or changes in communication and play (Miletic et al., 2024).

#### What Recommendations Are Listed for Child and Family Involvement in Interventions from SLP Association or Regulatory Body Documents?

All documents discussed family-centered care as a key tenet of SLP practice, including principles such as being aware of each family’s capacity to take on the demands of interventions, considering the heterogeneity and individuality of autism, ensuring that interventions are an agreed upon choice for the autistic person, and tailoring interventions to each family’s strengths, preferences, and priorities (RCSLT, 2023; SAC, 2023; SPA, 2022). Family-centered care involves consideration for each family’s environment; multiple documents outlined the importance of teaching family members how to better understand autistic people’s communication and considering how the physical environment impacts communication and participation (RCSLT, 2023; SAC, 2023; SPA, 2022).

## Discussion

The values of strength-based and family-centered services are recommended in SLP research, practice, and professional association/regulatory body documents related to autism. Yet these principles were not consistently reflected in the 26 SLP social communication interventions for autistic children and youth identified in our scoping review. Most studies described only deficits associated with autism; only three mentioned strengths associated with autism. Children’s perspectives were rarely included to inform intervention activities and were never included to inform intervention goals or outcome measurement. Parents’ perspectives were included for most interventions but often reflected choices from options offered rather than intervention decisions.

### Unpacking and Challenging Deficit-Focused Approaches to Autism

The deficit-focused descriptions of autism found in SLP social communication intervention literature were not surprising, as they align with predominant medical model approaches in healthcare and autism diagnosis, where each sentence of the autism diagnostic criteria begins with “deficits in . . .” (APA, 2013). Two of the three studies describing autistic people’s strengths were published in 2024, which may indicate evolving conceptualizations of autism. However, other recent studies focused on deficits, underscoring continued variability in approaches to autism. Most studies used person-first language, which aligns with a medical perspective that views autism as a condition that someone has, rather than a part of their identity (Bottema-Beutel et al., 2021; Kenny et al., 2016; Taboas et al., 2023). No studies explicitly mentioned consultation with families about terminology use or explained their rationale for using person or identity-first language. Many studies used dichotomous labels such “high- or low-functioning” or “verbal” and “non-verbal.” These labels are viewed as harmful by many autistic people due to their potential to reduce autistic people’s abilities to binary categories and obscure individualized needs and strengths (Alvares et al., 2020; Bottema-Beutel et al., 2021; Bottema-Beutel et al., 2025).

It is necessary to challenge deficit-focused approaches to autism to ensure that interventions meet the self-reported needs of autistic people and are consistent with strength-based and neurodiversity-affirming principles that are recommended in the field of SLP (Boron, 2025; Donaldson et al., 2017; Gaddy & Crow, 2023; Timler & McCaslin, 2025). The SLP professional association/regulatory body documents we analyzed mirrored the diversity in perspectives, recommendations, and language related to autism found in the field of SLP. Most documents explicitly opposed speech-language pathologists “treating” autism or framing social communication differences as deficits (RCSLT, 2023; SAC, 2023; SPA, 2022), while others left to practitioners and families the choice to frame autism as a deficit or difference (ASHA, 2025). The SLP documents also commented on potential reasons behind deficit-focused language, stating how a medical model approach may be needed to facilitate service access (ASHA, 2025; RCSLT, 2023).

SLP researchers and practitioners striving for strengths-based autism services must consider the driving factors behind current medicalized approaches, such as systems that determine who receives a diagnosis, eligibility for services, and funding for services. For example, practitioners may work with autistic children, youth and families in a strengths-based manner but write about a child’s deficits in a report to demonstrate service need or eligibility (Boyd et al., 2025). Practical ways to implement a strength-based approach while demonstrating the need for services need can include writing about how services are required to create supportive physical and social environments (e.g., fund a communication device, one-on-one aide to support in the classroom, parent coaching), rather than framing those needs as deficits within the child (Rosenbaum, 2021). SLP researchers and practitioners can also advocate for change at the systems-level to shape policy, funding, and programs offered so that systems can be more family-centered and strength-based (ASHA, 2016; SAC, 2016).

### Considerations for Delivering Neurodiversity-Affirming Social Communication Services

SLP clinicians and researchers must challenge long-held medical approaches to autism and rehabilitation to align with neurodiversity-affirming, rights-based approaches to intervention (Davies, 2024; Gaddy & Crow, 2023; Gibson et al., 2015; Hammell, 2015). Kapp (2020) describes the nuanced relationship between social and medical models of disability in healthcare service provision, describing how the neurodiversity movement generally disagrees with certain intervention approaches, yet it supports developmental therapies to build flexibility, language, and self-advocacy skills in a strength-based manner without “framing these matters in unnecessarily medical or clinical ways” (Kapp, 2020, p. 8). Australia’s guideline for supporting autistic children and their families is one example of a national document providing guidance on delivering social communication services. The guide encourages practitioners to consider the impact of their social communication goals on a child’s identity and document practices to safeguard the child’s right to preserve their identity (Trembath et al., 2022).

One example of how practitioners can provide neurodiversity-affirming social communication services is talking about autistic characteristics in a descriptive manner and acknowledging how the same trait can be a strength, challenge, or neutral depending on the social context. For example, a “failure of normal back-and-forth conversation” (APA, 2013) can be re-framed as a communication difference. The ability to share detailed information about topics of interest can be a strength when communicating with someone familiar or about a specific topic, but a challenge when meeting new people. Neurodiversity-affirming SLP goals could include self-advocacy about communication needs and preferences or learning about the double-empathy problem (Milton, 2012). We direct interested readers to explore neurodiversity-affirming approaches to SLP social communication intervention (Santhanam, 2023; Timler & McCaslin, 2025), and tools such as the Autism Understanding Tool for Introspection and Evaluation (AUTIE) to examine speech-language pathologists’ attitudes toward autism (DeThorne et al., 2024).

Another tension that may arise is balancing family-centered care and neurodiversity-affirming practices. Family-centered care and neurodiversity-affirming care share philosophies of centering autistic people’s expertise, recognizing the individuality of each family’s experience, and strength-based care (Dunst & Espe-Sherwindt, 2016). However, some autistic people may prefer person-first language or come from cultural backgrounds where autism and neurodiversity are conceptualized differently (Bruno et al., 2025; Rivera-Figueroa et al., 2022; Stahmer et al., 2019). There is increasing recognition that current conceptualizations of neurodiversity-affirming care are not universally applicable and often center white, Western perspectives (Nair et al., 2026; Srinivasan, 2025). One example of how to deliver services in a family-centered, neurodiversity-affirming manner involves practitioners asking about and honoring each family’s terminology preferences (e.g., person- or identity-first), while also sharing why many autistic people prefer identity-first language, including resources from autistic self-advocates (e.g., Garvey, 2024). Srinivasan (2025) provides excellent examples of tensions in the neurodiversity movement and how researchers and clinicians can address these challenges in a culturally responsive, family-centered way.

SLP researchers and clinicians can also consider if their social communication interventions promote camouflaging (i.e., masking and impression management), either directly or indirectly. Camouflaging is used by some autistic people, to consciously or unconsciously manage their impressions in high-stakes settings (e.g., job interviews) or lessen stigma in social environments (Finn et al., 2023), but has been connected to negative mental health impacts (Bradley et al., 2021; Cage, Troxell-Whitman, 2019; Cassidy et al., 2020; Pearson & Rose, 2021). To provide family-centered, neurodiversity-affirming care, clinicians can deepen their own understanding of camouflaging and discuss it with autistic clients and families, using accessible resources (e.g., Garvey, 2024; Duffus, 2023; Sedgewick et al., 2021). Practitioners can support clients to develop self-awareness of camouflaging behaviors, including when and why they choose to camouflage, and discuss the importance of embracing their authentic social communication preferences when, where, and with whom they feel safe to do so (National Autistic Society, n.d.).

Any conversations about masking must consider intersectionality, including what camouflaging looks like in different cultures (e.g., individualist or collectivist societies), or how client, family, and community values and culture may shape camouflaging behaviors across contexts (e.g., at home, at large family gatherings, in public spaces). Practitioners approaching conversations on camouflaging must also consider how systems of privilege and oppression (e.g., racism, classism, cisgenderism, and sexism) intersect with ableism to shape experiences of autism and make masking necessary for many visible minorities in society (Cage, Troxell-Whitman, 2019; Nelson & Lichwa, 2025; Pearson & Rose, 2021).

### Parent and Child Input on Interventions: Who Is Asked and How?

Engaging children and families in intervention decisions is an integral component of family-centered care (Bamm & Rosenbaum, 2008; Law et al., 2005). We did not find any examples of children’s perspectives included in intervention goals or outcome measurement and only two studies incorporated children’s activity preferences. We echo calls to include the first-person perspectives of autistic children, and not to rely solely on proxy reports from parents or professionals (Karni-Visel et al., 2024; Tesfaye et al., 2019). There are many established approaches to obtain children’s perspectives in research, including art-based methods (e.g., draw-and-tell, photovoice), visual supports in interviews, and other techniques that look beyond solely linguistic communication to include movement or gestures (Gonzalez et al., 2025; Scott-Barrett et al., 2022). Including autistic children’s perspectives on social communication interventions enables meaningful insights into their lived experience and can help ensure that services are tailored to children’s goals, interests, and motivations (Hummerstone & Parsons, 2022).

Although most studies sought some parent input, involvement often reflected intervention choices, rather than major decisions. For example, parents often chose intervention activities or goals within pre-set intervention aims and protocols or were asked for feedback on intervention outcomes in interviews or questionnaires. Selecting choices within a pre-set intervention purpose does not mean that those goals are important to the child and family, and standardized test score changes should not be extrapolated to reflect meaningful participation changes. One example of a measure that focuses on communication participation is the Focus on the Outcomes of Communication Under Six (FOCUS), which is a validated tool that looks at “real world” changes in communication following intervention and can be used in research and clinical practice (Thomas-Stonell et al., 2010). SLP clinicians and researchers can involve families in decision-making by exploring parents’ expectations of therapy early on (Phoenix et al., 2020), explaining intervention options throughout therapy, and framing the therapeutic relationship as one of partnership to reduce power imbalances (Auert et al., 2012).

Clinicians and researchers interested in collaborative goalsetting methods may wish to explore Goal Attainment Scaling (GAS), which was used in some studies in our review (Adams et al., 2020; Pereira et al., 2022). SLP researchers and clinicians can apply the double empathy problem to goalsetting by considering how the communication partners (e.g., friends and family) of autistic people can work toward communication goals, not only the autistic person (Milton, 2012; Milton et al., 2022). Communication partner training is one established intervention method in other areas of SLP that could be applied to social communication interventions for autistic youth and their communication partners (Albin et al., 2024; Anderson et al., 2024).

Finally, we did not find any examples of methods such as co-design or participatory action research being used. Autistic people are often left out of research or engaged in inequitable ways (Bertilsdotter Rosqvist et al., 2019; Cage et al., 2024; Fletcher-Watson et al., 2021), and future research should strive toward meaningful engagement and co-creation (Moll et al., 2020; Nicolaidis et al., 2019). Co-creation aligns with emancipatory autism studies, as co-creation principles center autistic people as experts in their own experience and seek to repair historical devaluation of autistic people’s knowledge compared to that of researchers who are mostly neurotypical (Bertilsdotter et al., 2019; Fletcher-Watson et al., 2019; Milton, 2014). We did not find any examples of co-design in the extant SLP social communication intervention literature for autistic people and their families, but there is an growing body of work using co-design methods with autistic children and youth in areas such as mental health (Cullingham et al., 2024), peer support (Davies et al., 2024), and education on sensory preferences (Hummerstone & Parsons, 2021).

### Limitations and Future Directions

First, our authorship team did not include autistic people in conceptualization or analysis; their inclusion may have led to different perspectives brought to data analysis or research questions being asked. Our work does represent the perspectives of an interdisciplinary, Canadian team of healthcare professionals and researchers (e.g., speech-language pathologists, an OT, and a pediatrician) who have experience with social communication services for autistic children, youth and their families. Second, most studies in our review focused on young children, with only one study including children over age twelve. Our results should therefore be interpreted as related to younger autistic children and may not apply to older children and youth. This age distribution is consistent with autism research focusing primarily on younger children (Jang et al., 2014), and the predominant emphasis on “early intervention” in autism (Fuller & Kaiser, 2020; Maksimović et al., 2023). Third, our translation to English for the gray literature was limited to automatic translation on Internet browsers. We did not have access to a professional translator, and it is possible that we missed relevant gray literature that did not translate accurately into English.

Fourth, we may have missed some relevant sources on SLP social communication interventions due to the vague nature of how social communication is operationalized (Tajik-Parvinchi et al., 2021). We relied upon authors’ labeling of interventions as social communication-focused and did not make judgments about how authors operationalized the concept of social communication. We used a variety of terms to refer to social communication in our search, and the language referring to social communication varied widely within and between articles. Some interventions focused on conversation, while others focused on language and AAC (Lerna et al., 2014; Lerna et al., 2012) or executive-functioning skills (Sun et al., 2017). The breadth in what studies meant by social communication mirrors the wide variety in how the concept of social communication is understood and defined (Tajik-Parvinchi et al., 2021) however studies that are more focused on a particular aspect of social communication (e.g., conversation skills) may choose more narrow operationalization. Finally, we reported how authors in research studies talked about autism and how they described child and family involvement in interventions, however these reports may differ from what occurred with children and families for a variety of reasons (e.g., limited word count, relevance to that study’s focus). Future research on client and families’ experiences of services, how speech-language pathologists view their services, and what strength-based and family-centered “ingredients” are part of how speech-language pathologists deliver social communication interventions would be valuable (Van Stan et al., 2019). A future direction for speech-language pathologists is to reflect on how individual factors (e.g., attitudes and actions in therapy sessions) and systemic factors (e.g., funding models, diagnostic process, and environmental accommodations) impact service delivery, and what needs to change to move toward more strength-based, family-centered, and neurodiversity-affirming services.

## Conclusion

Clinicians and researchers may find our analysis of SLP social communication interventions useful as a basis to reflect on their own intervention practices, including how they describe and understand autism, how children and family perspectives are incorporated in interventions, and how interventions align with strength-based and family-centered care principles. Our review outlined how SLP social communication interventions already incorporate parent perspectives in goals, activities, and outcome measurement, and described several suggestions for advancing child and family involvement toward shared decision-making. SLP researchers and clinicians can engage autistic people and their families as key decision-makers in intervention research and delivery, describe autism and autistic children in a strength-based manner, and consider how their intervention approaches align with current SLP regulatory body and professional association guidelines. This scoping review adds to the body of literature on social communication interventions for autistic people in the field of SLP by analyzing whether and how the strengths and perspectives of autistic children, and their families, are incorporated.

## Supplemental Material

sj-docx-1-aut-10.1177_13623613261448948 – Supplemental material for How Do Speech-Language Pathology Social Communication Interventions Incorporate the Strengths and Perspectives of Autistic Children and Their Families: A Scoping Review Supplemental material, sj-docx-1-aut-10.1177_13623613261448948 for How Do Speech-Language Pathology Social Communication Interventions Incorporate the Strengths and Perspectives of Autistic Children and Their Families: A Scoping Review by Maya Albin, Peter Rosenbaum, Eniola Bode-Akinboye, Yani Hamdani and Michelle Phoenix in Autism

## Notes

1. Identity-first language is used throughout this paper as this terminology is preferred by many in the autistic community, reflecting that autism is “inseparable from and fundamental to an individual’s experience of the world” (Bottema-Beutel et al., 2021, p. 21; Taboas et al., 2023).
