# Supplementary material for: How Do Speech-Language Pathology Social Communication Interventions Incorporate the Strengths and Perspectives of Autistic Children and Their Families: A Scoping Review
Source: Autism. 2026 Jun 3;30(7):1663–86. doi: 10.1177/13623613261448948 (PMC13287380; doi:10.1177/13623613261448948)
Supplement: sj-docx-1-aut-10.1177_13623613261448948 – Supplemental material for How Do Speech-Language Pathology Social Communication Interventions Incorporate the Strengths and Perspectives of Autistic Children and Their Families: A Scoping Review [file sj-docx-1-aut-10.1177_13623613261448948.docx]

**Supplementary Material 1.**

*Sample Search Terms from OVID Medline Database*

| Category | Subject Headings | Keywords |
| --- | --- | --- |
| (Population) | Autism Spectrum Disorder/  Autistic Disorder/ | autis*.mp.  ASD.mp.  Asperger*.mp.  PDD NOS.mp. |
| (Population) |  | Child*.mp.  Youth.mp.  Toddler*.mp.  Teen*.mp.  Adolescen*.mp.  Pre-teen*.mp.  Pre-adolescen*.mp.  Kindergarten*.mp.  Preschool*.mp. |
| (Concept) | Social Skills/  Social Interaction/  Social Behaviour/ | social* competen*.mp.  Social* communicat*.mp.  Social* skill*.mp.  Pragmatic*.mp.  Social* interact*.mp.  Social cognition.mp.  Social interven*.mp.  Social* relat*.mp.  Social cue*.mp.  Social behav*.mp. |
| (Context) | Speech Language Pathology/  Speech Therapy/ | Speech lang*.mp.  Speech thera*.mp.  SLP.mp.  SLT.mp.  Language ther*.mp.  Communication ther*.mp. |

Terms in each category were connected with ‘OR’, and each group was connected with ‘AND’

* = truncation, / = subject heading

**Supplementary Material 2.**

*List of Speech-Language Pathology Professional Associations Searched*

| Professional Association Name | Country |
| --- | --- |
| Speech-Language Audiology Canada (SAC) | Canada |
| American Speech, Language and Hearing Association (ASHA) | USA |
| Asociacion Argentina de logopedia Foniatria y Audiologia (ASALFA) | Argenina |
| Sociedade Brasileira de Fonoaudiologia | Brazil |
| Speech-Language and Audiology Association of Trinidad and Tobago (SLAATT) | Trinidad & Tobago |
| Speech Pathology Australia (SPA) | Australia |
| Chinese International Speech-Language and Hearing Association (CISHA) | China |
| The Hong Kong Association of Speech Therapists (HKAST) | Hong Kong |
| The Korean Academy of Speech-Language Pathology and Audiology | Korea |
| Malaysian Association of Speech-Language and Hearing (MASH) | Malaysia |
| New Zealand Speech-Language Therapists Association (NZSTA) | New Zealand |
| Saudi Society of Speech-Language Pathology and Audiology | Saudi Arabia |
| Speech-Language & Hearing Association Singapore (SHAS) | Singapore |
| The Speech-Language-Hearing Association, Taiwan | Taiwan |
| Egyptian Society for Phoniatrics and Logopedics | Egypt |
| South African Speech-Language-Hearing Association (SASHLA) | South Africa |

**Supplementary Material 3.**

*Data on Social Communication Interventions*

| Citation | Intervention name and any acronyms | Manual-ized? | Professionals delivering the intervention^a^ | Intervention aims (A)^b^ | Intervention outcome measures (O)^b^ |
| --- | --- | --- | --- | --- | --- |
| Adams et al. (2020) | Social Communication Intervention Program (SCIP) | Y | SLTs + “special needs teacher” | Only discusses previous study (not current study) – “**carer-rated** **pragmatic competence** and **changes in social communication and language skills**, teacher-rated learning skills and an observational measure of conversation skills” p. 2 | “Parents provided three priority areas for intervention at baseline assessment (time 1). In discussion with the RSLT and practitioner, three goals for the SCIP intervention period were set to reflect these priorities. The **SCIP-GAS form** set out the parent priority, the baseline level of ability and the goal (desired ability) after an intervention” p. 4 |
| Carter et al. (2011) | More Than Words (MTW) | Y | SLPs only | Focuses on “helping children reach the following four goals: (a) improved **two-way interaction**, (b) more **mature and** **conventional ways of communicating**, (c) better skills in **communicating for social purposes**” p.744 | (a) Parent Interview for Autism-Clinical Version (PIA-CV): Measures autism symptom severity, with the **Nonverbal Communication domain** analyzed for child communication outcomes.  (b) The Early Social Communication Scales (ESCS): Evaluates nonverbal communicative behaviors, focusing on **Initiating Joint Attention (IJA)** and **Initiating Behavior Requesting (IBR).**  (c) The Parent-Child Free Play Procedure (PCFP): Observes child and parent interactions, measuring **parental responsivity and child intentional communication**. |
| Casenhiser et al. (2013) | Milton & Ethel Harris Research Initiative Treatment (MEHRIT) Program | NR | SLPs or OTs | “To improve children’s **social interaction and communication abilities**… focus the investigation on four factors important to social interaction …**quality of social interaction**…ability to engage in and initiate **joint attentional frames**...degree to which child seems to **enjoy interacting with caregiver**...language ability" p. 222 | “Rate children’s interactions with their parents at 0 and 12 months into treatment. The 5-point Likert-type scale is designed to track what Mahoney and colleagues call **‘pivotal behaviors’**" p. 226 |
| Chang et al. (2016) | Joint Attention, Symbolic Play, Engagement and Regulation (JASPER) adapted for classroom | Y | SLPs + SLP assistant, behaviour consultant, special education teacher, teaching assistant | “Improve children's **joint attention**, **joint engagement and play skills** in the classroom”  p. 2212 | (a) Ten-minute teacher-child play interactions (TCX): coded for the primary teacher outcome (teachers’ strategy implementation) and primary child outcomes (duration of **joint engagement,** frequency of **initiated joint attention/**initiated behavioral regulation**,** **diversity of play skills);** (b) Early Social Communication Scales: used to assess **joint attention** and behavior regulation skills. Coded videotapes for the type and frequency of **spontaneous joint attention skills (IJA)** and spontaneous behavior regulation skills (IBR); (c) Structured Play Assessment, coded “the **number of unique spontaneous play types and frequency of those acts** were coded based on 16 levels of play ranging from indiscriminant to thematic play” p. 2216 |
| Divan et al. (2015) | Parent-mediated-intervention for Autism Spectrum Disorder in South Asia (PASS), adaptation of Preschool Autism Communication Therapy (PACT) | Y | SLP originally, adapted for other disciplines (OT, developmental pediatrician, psychiatrist) | "PACT strategies act to make specific and theoretically based alterations in a child’s dyadic communication environment in order to **improve the child’s social communication,** attention, and language**”** & “Original UK PACT follows a developmental approach to supporting communication, addressing social, pre-linguistic, pragmatic, and linguistic impairments which are present in ASD" p. 2 | N/A – all outcomes related to cultural adaptation, not intervention aims |
| Franco et al. (2013) | Prelinguistic Milieu Teaching (PMT) | Y | SLP + BCBA | "To increase children’s use of **intentional prelinguistic communication** skills” p. 490 | "All of the **intentional communication acts**, defined as any attempt that the child made to interact with the adult within the social routine using vocalizations, gestures, or eye gaze, were coded. Coding included the time of the communication act, ordinal sequence of the communication act within the routine, initiation by child or adult, form of communication (i.e., vocalization, gesture, and/or eye gaze) used, and whether each form was spontaneous or prompted" p. 494 |
| Girolametto et al. (2007) | More Than Words (MTW) | Y | SLPs only | “The first aim of this study was to confirm that **parents used responsive interaction strategies following intervention**…The third and most important aim of the study was to examine the children’s **social interaction skills** following intervention, specifically their **rate of communicative acts**, participation in **social interaction sequences**, and **initiation of social interaction**" p. 475 | Many outcome measures (p. 478-479), including (a) coding parent interactions for use of specific behaviours, (b) The Joy and Fun Assessment (JAFA) to assess parents’ use of **responsive interaction during play** with nine parental strategies rated, (c) child vocabulary size (d) lexical diversity and rate of communication acts, (e) parent **engagement in social interaction sequences** (“identified reciprocal social interaction sequences that were defined as chains of parent-to-child turns”), (f) **children’s initiation of social interaction**, (g) parent report of program progress |
| Godoy et al. (2024) | Paediatric Autism Communication Therapy (PACT) | Y | SLPs + other disciplines (OT, medicine, psychology) | "Support child development via **increasing parental sensitivity and responsivity** to the autistic child’s communication and interaction as well as **improving parental understanding** of autistic and non-autistic social-communication development more broadly and their child’s social-communicative behaviour specifically” p.124 | Interviews discussing “general impressions about having received PACT (benefits, harms and challenges)…**changes in the dyad’s interaction behaviour**” p. 130 |
| Green et al. (2010) | Preschool Autism Communication Trial (PACT) | Y | SLTs only | "The aim of the intervention was first to increase parental sensitivity and responsiveness to child communication and reduce mistimed parental responses." (p. 2153) | (a) “primary outcome was the ADOS-G **social**  **communication algorithm score** — a measurement of the severity of the symptoms of autism". Secondary outcomes including (b) **parent-child interaction** during naturalistic play in a standard (non-therapy), (c) child language and social communication with several measures including Communication and Symbolic Behaviour Scales Developmental Profile (CBS-DP, caregiver questionnaire) **social composite raw scores** (d) adaptive functioning in school beyond the family" p. 2154 |
| Hutchins & Prelock (2006) | Social Stories & Conversation Comic Strips | NR | SLPs supervising SLP graduate students | “[Social Stories] are designed to  **minimize those factors identified as potentially confusing** during interaction to provide individuals with autism “direct access to social information”…[Conversation Comic Strips] are designed to **improve social interaction** by facilitating joint attention and shared meaning-making” p. 49, "The goal of these particular interventions was to **promote perspective-taking skills** and to **reduce the frequency of a recurring social conflict**." p. 53 | Narrative description of changes from A-B for case study, not specifically aligned with aims (no formal measures) |
| Hutchins et al. (2013) | Social Stories | NR | SLPs only | “Promote positive change in behavioural and **communicative functioning**…sought to identify a communicative impairment (e.g., difficulty greeting others or maintaining eye-contract during conversation) to construct a communication story with the aim of **enhancing a particular** **aspect of** **communication**” p. 384 | Diaries of "parents’ daily ratings of the behavioural and **communicative functions targeted during intervention**" (e.g., Joseph, "very social today. Maybe well rested? ”, “ More social”, “Getting better”) p. 385 |
| Katz et al. (2013) | Peer mediated intervention program | NR | SLP + ECE | “Whether, following the intervention, peer interventionists and target children with ASD engaged in **more frequent and longer extended social interactions**…whether lay independent observers think that the intervention achieved an important social goal, defined as **increases in the number and length of extended interactions**" p. 134 | "Baseline data were collected for each child in interaction with each of the two typically developing peers, in two naturalistic play contexts (i.e., play dough, block play)” p. 136, "Pre- and Postintervention Ratings on the  **Social Interaction Assessment Scale**" p. 140 |
| Lerna et al. (2012) | Picture Exchange Communication System (PECS) | Y | SLPs only | “Test the effects of the first four PECS  phases on **social–communicative behaviours** of children with ASD” p. 611 | “The efficacy of treatment was assessed via a battery of **standardized assessments of social–communicative abilities**. In addition, several behavioural measures were derived from observation of a free-play session with an examiner” p. 612 |
| Lerna et al. (2014) | Picture Exchange Communication System (PECS) compared to Conventional Language Therapy (CLT) | Y | “Specialists in SLP with extensive expertise with ASD” | CLT "treat language and communicative disorders in developmental disabilities in accordance with the guidelines of the Italian National Health System"; PECS: "**enhance social and communicative skills (e.g., initiations, requests, joint attention, social interaction with peers and turn-taking)** in ‘nonverbal’ children with autism” p. 479 | (a) Griffith Mental Developmental Scales Language, **Personal-Social** & Nonverbal-IQ subscales, (b) ADOS “**communication, reciprocal social interaction** and total scores were used to assess change over time”, (c) Vineland Adaptive Behavior Scales II (VABS) gathered parent report of the **child’s communication and social abilities**; (d) Unstructured Free Play with examiner “**social-communicative measure**s (i.e., joint attention, verbal and nonverbal requests, initiation, cooperate play and eye contact) operationalized |
| Lim et al. (2007) | Pilot clinic-based social skills group | NR | SLP & OT co-facilitating | Improve the child’s (a) **interpersonal social skills** (e.g., make friends, greet friends, play and interact appropriately) (b) **learning-related** **social skills** (e.g., concentrating on tasks, following instructions, working in pairs or groups) and (c) equip parents with goals, strategies, and activities to continue **social skills development** well after the child has completed the 8 weekly intervention sessions. | (a) Observation Profile to assess reduction from pre- and post-test scores for **social interaction, social communication, social imagination**…(b) Parent Perception Form – “asked to give specific examples of improvement in their child after attending the group” related to **social skills** p. 36 |
| Macevilly et al. (2024) | Secret Agent Society (SAS) | Y | SLPs only | “Determine if the SAS programme led to improvements up to 6-months post-intervention in children’s (a) **social communication skills** at home and at school; (b) **emotional regulation** skills at home and at school” p. 58” | Several forms completed by parents, teachers and children at four discrete timepoints: (1) **Social Skills** Questionnaire (SSQ) parent and teacher versions, (2) **emotion regulation** **and social skills** questionnaire (ERSSQ) parent and teacher versions” |
| Miletic et al. (2024) | More Than Words (MTW) | Y | SLPs only | “Designed to **support social communication and play development** of children 5 years old and under who have either an autism diagnosis or social communication difficulties" p. 1128 | Child Profile Part 1 – rated “based on The Hanen Centre’s four **social communication stages**… a) Own Agenda: the child sends messages unintentionally, (b) Requester: the child sends messages intentionally to request or protest, (c) Early Communicator: the child sends messages to connect with others for social purposes, and (d) Partner: the child has short conversations with others” p. 1128; "Final Reflection and Evaluation form… 10 open-ended questions across the following topics: (a) perceived changes in **interaction and/or communication in both caregiver and child**, (b) helpful/unhelpful aspects of the program, (c) changes perceived by people outside the family, (d) impact of the program on the family as a whole, (e) satisfaction with the SLP, and (f) suggestions for change/improvement" p. 1130 |
| Mohammadzaheri et al. (2022) | Pivotal Response Treatment (PRT) | NR | SLPs supervising SLP graduate students | "Investigate the impact of PRT on enhancing **verbal initiations**” p. 2599, intervention if targeting verbal social initiations would improve (a) **spontaneous question-asking**, (b) mean length of utterance, (c) broader improvements in untreated areas measured by a structured communication checklist | "Data were collected on **the number of initiations each child** made during the 30-min  unstructured play interactions…each child’s parent was asked to complete the Children’s Communication Checklist (CCC) to assess for gains in structural characteristics of **verbal interactions and pragmatic areas**." p. 2604 |
| Muller et al. (2016) | Conversation Club Curriculum | NR | SLPs + other disciplines (teacher, OT, social worker) | "Increase participants’ **spontaneous, meaningful, and naturalistic conversation** with peers" p. 193 | (a) operationalized five **observable conversational behaviors** to determine whether there was an increase in the frequency of these behaviors between baseline and post- intervention; (b) interviews with the participants’ SLP regarding her impressions of participant-specific **growth in terms of nine conversational skills**; (c) thematic analysis of video footage gathered during baseline, active instruction, and postintervention in order to describe **characteristics of participants’ conversation** at each point in time; (d) to establish social validity …conducted interviews with the five members of the Conversation Club intervention team regarding their overall impressions of program effectiveness" p. 196 |
| Parsons et al. (2018) | Peer to peer play based intervention | NR | SLP or OT | “This intervention focuses on promoting **positive**  **dyadic interactions** between playmates during  cooperative social play, the initial interactive process that children engage in with each **other in order to develop and maintain friendship**" p. 413 | (a) The Pragmatics Observational Measure (POM): evaluates skill level and consistency on a four-point scale **for five pragmatic domains**…language domains…introduction and **responsiveness**…non-**verbal communication**…**socio-emotional attunement**…executive function…negotiation" p. 415. (b) Social Emotional Evaluation (SEE), “child’s ability to understand and explain the **social cues** of others” p. 416, (c) Profiling Elements of **Prosody** in Speech Communication (PEPS-C) |
| Pereira et al. (2022) | Pragmatic Intervention Programme (PICP) | Y | SLTs only | “Aims to promote **pragmatic language skills** among preschool-age children with pragmatic impairments”, skills: (1) **eye contact**, (2) **joint** **attention**, (3) **turn-taking**, (4) **communicative response**, (5) **communicative initiative**, (6) **communicative functions**, (7) comprehension and expression in verbal and non-verbal communicative contexts, (8) cohesion, (9) inferential comprehension, (10) **conversation**, and (11) **figurative language** and advocates that these skills should be worked on with different communicative partners (e.g., peers, teachers) and in multiple contexts (e.g., home, kindergarten) to promote skills generalization" p. 2 | (a) Goal Attainment Scale "child’s needs and the parents’ and kindergarten teachers’ priorities were mapped on to the skills addressed in the PICP and then three appropriate goals were jointly selected " p. 4. Secondary outcomes (b) EACC, an assessment scale used to evaluate the family’s and teachers’ **perceptions of the child’s pragmatic skills**, specifically in the following areas: (I) communicative intentions; (II) conversational skills; (III) responsiveness in communicative contexts; (IV) comprehension in communicative contexts; (V) coherence; (VI) cohesion; (VII) non-literal language comprehension; and (VIII) extralinguistic aspects |
| Salt et al. (2001) | Scottish Centre for Autism Preschool Treatment Programme | NR | SLPS + other disciplines (OTs, teachers, nursery nurses) | “Aim of therapy is to help the **child become more flexible and accessible to learning how people communicate.** We look to increase the **child's motivation for reciprocal social contact** by improving the child's **non-verbal social communication, language, imitation and social interaction skills**. These abilities can then lead to the subsequent development of play, flexibility of behaviour and necessary preschool social skills. We hope to help the child be in a better position to make use of educational opportunities available to them in the future" p. 366 | N/A – program report without any outcome measures |
| Shire et al. (2020) | jasPEER, adaptation of Joint Attention, Symbolic Play, Engagement and Regulation (JASPER) | Y | SLPs + other disciplines (social workers, OTs) | Aims “to advance children’s individual social communication and play skills as well as support peer engagement …would children in jasPEER demonstrate greater change in **unsupported peer engagement** then children in classrooms receiving JASPER? Finally, would children in JASPER classrooms show **greater changes in social communication and play skills** than children randomized to jasPEER classrooms" p. 2143 | (a) Teacher–child interaction… coding for TA's strategy implementation and children's joint engagement; (b) SPACE, a "tool designed to capture children’s **spontaneous initiations of joint attention** (IJA) and behavioral regulation (IBR), as well as children’s **spontaneous play skills** by type and level"; (c) non-adult-mediated Peer Interaction Observation p. 2147 |
| Sun et al. (2017) | Simulation of Executive Functions (SEF) | NR | SLPs only | “We aimed to observe if the inclusion of tasks involving [Executive Functions] in the language intervention process with children with autism may be beneficial as observed by improved performance in areas of **functional communication** and **social cognitive performance"** p. 80 | "All participants were assessed regarding the Functional Communicative Profile (FCP) and the Social Cognitive Performance (SCP) in the pre- and post-intervention periods implemented", operationalized how they defined improvement in FCP and SCP, p. 80 |
| Van Der Meer et al. (2014) | Preference-enhanced communication intervention | NR | SLP + support worker | "To determine if a modified behavioral intervention with Ian’s most preferred AAC system would result in **more spontaneous, complex, and socially oriented communication**." (p. 284) | Measures of "**self-initiated requests**, responses after verbal cues, incorrect responses, and no response" (p. 287), “percentage of **response types** across communicative categories" (p. 288 |
| Williams et al. (2024) | Music Assisted Programme (MAP) | NR | SLTs only | MAP: Aims not listed, MAP protocol stated "parents were taught strategies including intensive interaction and communication temptations to **help them engage with their children**" p. 2519. “SCIP-I group, sessions were given focusing on **social communication strategies** with focused stimulation of the target words" p. 2517 | Social Responsiveness Scale (SRS)-2 & "Social communication was evaluated from a 10-min sample of free play between the child and their parent recorded by the parent at home" coding for **social responses** & **social initiations** p. 2520 |

*Notes.* NR = not reported; Y = Yes; N = No; N/A = not applicable. SLP = speech-language pathologist; SLT = speech-language therapist; OT = occupational therapist; BCBA = Board Certified Behaviour Analyst; ECE = Early Childhood Educator.

^a^We included interventions require SLP involvement either in carrying out the intervention, supervising those carrying out the intervention, or developing the intervention. SLP involvement had to be listed in some capacity for an intervention to be included

^b^We extracted intervention on intervention aims and outcomes that were specific to the social communication aspects of the intervention. Intervention aims were extracted, which were not necessarily the same as study aims (e.g., a study focuses on evaluating cultural adaptation, but also discusses intervention aims – only intervention aims were extracted). For outcome measures, only social communication specific outcome measures were extracted (e.g., outcome measures related to vocabulary for children, SLP experiences delivering intervention would not be included).
